# Migration Evolves in Response to Distinct Regimes of Climate Seasonality in Tropical Versus Temperate‐Breeding Suboscine Birds

**DOI:** 10.1002/ece3.71745

**Published:** 2025-07-09

**Authors:** Emily X. Johns, Oscar Johnson, Michael G. Harvey

**Affiliations:** ^1^ Department of Biological Sciences University of Texas at El Paso El Paso Texas USA; ^2^ Department of Biological Sciences Florida Gulf Coast University Fort Myers Florida USA

**Keywords:** climate change, evolutionary biology, migration ecology, phylogeography

## Abstract

Avian migration has long captured human interest, but the causes of the evolution of migration remain unclear due to limited study of the full spectrum of migratory strategies, including short‐distance and intratropical movements. We examine the climatic drivers of migration across the roughly 1300 species of suboscine birds, a group containing many intratropical migrants. Comparative analyses confirm that migratory behavior in temperate‐breeding suboscines evolves in association with temperature seasonality. The evolution of migration in the tropics, however, has a more complex association with climatic variables including precipitation and greenness seasonality. Projections under future climate scenarios show that suboscines will experience average lower temperature seasonality, potentially favoring the loss of migration, but higher precipitation seasonality, potentially favoring an increase in short‐distance migration. The divergent impacts of climate seasonality on the evolution of different migratory strategies highlight the complexity of climate‐movement associations and the challenges of projecting responses to climate change.

## Graphical abstract

We used comprehensive data on phylogenetics, migratory behavior, and past and future climate to test the climate‐migration associations in a large radiation of roughly 1300 suboscine passerine birds. We demonstrate that while longer‐distance migration is influenced overwhelmingly by seasonal fluctuations in temperature, tropical migrations have more complicated associations with the environment, including precipitation seasonality and fluctuations in vegetation greenness. Our future climate projections (2080–2100) suggest that long‐distance, temperate‐breeding migrants will experience conditions that might reduce migratory propensity on average, while tropical breeders might experience conditions that either increase or reduce migratory propensity.

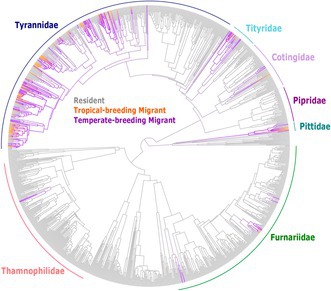

## 1 Introduction

Movement is a fundamental part of avian life history, shaping how birds acquire food resources (Gauthreaux 1982; Rappole 1995; Berthold 1996; Berthold 1999), avoid predators (Alerstam et al. 2003), and track favorable climate conditions (Winger et al. 2019). Migrations, or regular seasonal movements, are a key strategy for dealing with predictable events in many bird species—approximately 1800 of the 10,000 bird species worldwide undergo seasonal migration (Sekercioglu 2007; Rolland et al. 2014). Avian migratory behavior is important because it can drive geographical range dynamics and population expansions (Lester et al. 2007; Fritz et al. 2012), shape community assembly (MacArthur and Wilson 1967), regulate pollination and seed dispersal (Viana et al. 2016), spread zoonotic pathogens and diseases (Fèvre et al. 2006; Jourdain et al. 2007; Kobuszewska and Wysok 2024), and mediate speciation and persistence (Claramunt et al. 2012; Smith et al. 2014; Pegan et al. 2024). The drivers responsible for the evolution of migration, and how those drivers might lead to migratory changes in the future, are thus of fundamental interest.

The evolutionary acquisition of migration has long been attributed broadly to extrinsic factors such as climate and climate seasonality (Shaw and Couzin 2013). However, a detailed understanding of how climate influences migration is lacking (Åkesson and Hedeström 2008; Boyle et al. 2010; La Sorte et al. 2015; Zenzal et al. 2023). Ecological processes including competition for breeding and wintering territories (Cox 1968; Lack 1968; Voelker et al. 2013; Somveille et al. 2019), avoidance of predation (Boyle 2006), seasonally fluctuating food availability (Winger et al. 2019), and avoidance of climatic extremes (Nolan and Ketterson 1990) have been invoked to link migration to seasonal climates. However, progress in this area has been hampered by two challenges. First, both climatic and ecological drivers are difficult to parse because of high covariation among variables across species and geographic regions (Tablado et al. 2014; Zhang et al. 2017; Walker et al. 2020; Krenhardt et al. 2024). In addition, migration is not a simple binary variable but represents a complex array of movement strategies that may differ in their nature and origins (Boyle 2017; Evans and Bearhop 2022; Flack et al. 2022). Parsing this complexity may be key to distinguishing the relative roles of different drivers.

Long‐distance seasonal migrations represent one of the best‐studied phenomena in avian biology (Egevang et al. 2010; Winger et al. 2014; Conklin et al. 2021) and typically involve movement between a temperate breeding range and tropical wintering range. Short‐distance migratory movements, although widespread in birds, are comparatively poorly understood. Short‐distance migration might represent seasonal latitudinal movements between breeding and wintering grounds that are less distant than in long‐distance migrations, but an array of other forms exist (Hayes 1995; Barçante et al. 2017; Hsiung et al. 2018). Intratropical migrations include seasonal movement of birds that spend the entire year within the tropical latitudes (Stutchbury et al. 2016; Koleček et al. 2018; Jahn et al. 2019) or the movement of temperate‐breeding migrants between tropical sites during their non‐breeding period (Heckscher et al. 2011; Koleček et al. 2016; Stutchbury et al. 2016). They include latitudinal, longitudinal, elevational, and more complex patterns of movement (Gómez‐Bahamón et al. 2020). These forms of migration have been documented within Neotropical (Fraser et al. 2012; Heckscher et al. 2015), Afrotropical (Lemke et al. 2013), and Indomalayan/Australasian (Yong et al. 2015) migratory systems. Because many of these migratory strategies involve shorter and perhaps less conspicuous movements than long‐distance migrations and most migration research has taken place in the Global North, their diversity and distribution across the avian tree of life are incompletely known (Jahn et al. 2012). However, knowledge is improving, thanks in large part to local researchers and indigenous knowledge (Jahn et al. 2020; Jessen et al. 2022).

The suboscine passerines are a uniquely suited group of birds in which to study the interactions between climate and migratory complexity. Suboscines (Aves; Tyranni) comprise over 1300 species of perching birds distributed across both the Americas and the Old World tropics. Importantly, they exhibit a wide range of migratory strategies, including a high proportion of species that undergo intra‐tropical and short‐distance movements (Chesser 1994). We also know that suboscines occur in a wide array of climates and environments (Stotz 1996), and there is evidence that this climatic diversity has shaped the evolutionary success and diversity of the group (Harvey et al. 2020).

Here, we test how climatic seasonality factors influence suboscine bird species with different migratory behaviors. We ask the following: (1) How frequently have different migratory strategies been gained or lost across the evolutionary history of suboscines? and (2) Do different climatic regimes explain different migratory strategies? We use a comprehensive phylogeny of 1323 suboscines, data on migratory strategy, and high‐resolution climate data to conduct association tests and model causal relationships in a phylogenetic comparative framework. The results of these tests are key both to illuminating the mechanisms responsible for the evolution of migration and for providing a foundation for understanding how migration will continue to change under future climate scenarios.

## 2 Materials and Methods

### 2.1 Phylogenetic and Migration Data

For phylogenetic information, we used the primary, time‐calibrated tree from Harvey et al. (2020). This phylogeny was estimated using data from ultraconserved elements (UCEs) and conserved exons in a maximum‐likelihood framework and time‐calibrated using a penalized likelihood approach. It is well resolved and contains 97% of suboscine species, including both New World and Old World species.

Migratory behavior for each suboscine was extracted from the AVONET database (Tobias et al. 2022), in which migratory behavior for each species is coded as resident, partially migratory, or fully migratory. In AVONET, birds are classified as partially migratory if a minority of the population migrates long distances, if most of the population undergoes short‐distance migration, or if they undergo only nomadic movements or distinct altitudinal migration. Birds are classified as fully migratory if most of the population undertakes long‐distance migration, which is defined as having fully disjunct breeding and wintering grounds. We hereafter refer to this distance‐based strategy for coding migration as the “distance‐based coding”. We spot‐checked migratory information from AVONET to ensure accuracy.

Birds with temperate‐tropical migrations may differ in important ways from intratropical migrants. We therefore also classified migratory species as either temperate‐breeding or tropical‐breeding by determining whether the centroid of the breeding range of each migratory species was outside of the tropics (above 23.44° or below −23.44° latitude) or within the tropics. We refer to this strategy for coding migrants as the “latitudinal coding”. Bird species distribution maps were obtained from BirdLife International and Handbook of the Birds of the World (2022) and were used to analyze the geographic species densities of birds with different migratory strategies and to examine the climate histories experienced by each suboscine species. Only native distributions of birds were used.

### 2.2 Climate and Habitat Data

To measure historical climate trends across the globe, we obtained 18 bioclimatic variables and took the 2.5‐min resolution averages of data from 1970 to 2000 from WorldClim (Fick and Hijmans 2017). To understand the indirect effects of climate on migration, we obtained imagery for the monthly normalized difference vegetation indices (NDVI) for 2001, which were produced by the NASA Earth Observations (NEO) using data provided by the MODIS Land Science Team (NEO and MODIS Land Science Team 2001). We calculated an additional climate variable, “greenness seasonality”, by taking the normalized difference vegetation index (NDVI) values from 2001 and calculating the average annual range of NDVI over the distribution of each suboscine species.

We measured the 18 WorldClim Bioclimatic variables and greenness seasonality for all suboscine species by overlaying shapefiles of each breeding range onto the climate maps and calculating the mean, median, and standard deviation of each climate variable over the breeding distribution of each species. We conducted a suite of preliminary analyses of the WorldClim variables and greenness (Appendix S1, Table S1). Ultimately, we proceeded with BIO4 (temperature seasonality), BIO15 (precipitation seasonality), and greenness seasonality in focused association tests and causal modeling. These three variables best capture the climate variation thought to influence the evolution of migration (Studds and Marra 2011; Klinner and Schmaljohann 2020), and thus, their interpretation is relatively straightforward.

### 2.3 Evolutionary Reconstruction of Migration

Although migration in birds is quite plastic (Zink 2002, 2011), each re‐emergence of this trait reflects a potential independent response to extrinsic conditions that is a critical context for comparative analysis. We evaluated the history of migratory behavior in suboscines using an ancestral character estimation using the “ace” function in ape (Paradis and Schliep 2019). Using both distance‐based and latitudinal coding, we plotted the migration status onto the tips of the phylogeny using ggtree (Yu et al. 2017) and conducted stochastic character mapping of ancestral states using the “plotTree.wBars” function in phytools (Revell 2012). From these ancestral states, we quantified the number of independent origins and losses of migration on the suboscine tree, with partial and long‐distance migrants both classified as migrants (i.e., transitions between resident and migratory states across the tree). We also evaluated the phylogenetic signal in migratory behavior using Blomberg's K and Pagel's lambda in phytools (Revell 2012).

### 2.4 Comparative Analyses of Climate Seasonality and Migration

To evaluate potential causal factors responsible for the evolution of migration, we performed phylogenetic generalized least squares (PGLS) regressions on migration status (resident, temperate‐breeding, tropical‐breeding) versus BIO4 temperature seasonality, BIO15 precipitation seasonality, and greenness seasonality. For this PGLS, we utilized a Brownian correlation structure in Caper (Orme 2013), which performs a linear regression while accounting for phylogenetic covariation among species. R‐squared values for each regression were measured using the “R2_pred” function in rr2 (Ives and Li 2018).

We then used a phylogenetic ANOVA to determine the significance of the relationship between alternative migratory strategies and temperature seasonality, precipitation seasonality, and greenness seasonality using the “aov.phylo” function in geiger (Pennell et al. 2014). We used a phylogenetic path analysis (PPA) to analyze the relative direct and indirect contributions of these seasonality variables on the evolution of migration. PPA estimates the importance of alternative causal relationships among variables (Gonzalez‐Voyer and von Hardenberg 2014). A model set with 51 models describing possible interactions between environmental variables and partial and full migration was generated using the “define_model_set” function in phylopath (van der Bijl 2018). We then tested the fit of these models to our data and identified the model that best fit our data to produce a directed acyclic graph of the relative strength of the correlation between each of the three focal climate variables and short‐ (tropical‐breeding) versus long‐distance (temperate‐breeding) migration.

We also visualized the climatic niche of species according to their migratory behavior by plotting temperature seasonality versus precipitation seasonality across species. To simplify visualization, we categorized species as resident, temperate‐breeding migrants, and tropical‐breeding migrants for this analysis. We then explored the taxonomic variation in climate by species in each category.

### 2.5 Evaluation of Current Breeding Range Climates Under Future Climate Projections

To evaluate the influence of future climate change on species with different migratory strategies, we measured projected temperature seasonality and precipitation seasonality over the current breeding distributions of all suboscine species. We chose the MIROC 6 global climate model (GCM) and obtained 2.5‐min resolution maps of the 2 WorldClim Bioclimatic variables (BIO4 Temperature Seasonality and BIO15 Precipitation Seasonality), with the monthly values of each variable averaged over a 20‐year period: 2081 to 2100 (Eyring et al. 2016). These variables were measured over each distribution for two shared socio‐economic pathway (SSP)‐based climate scenarios (SSP 126 and SSP 370), which belong to a collection of one of five shared socio‐economic pathways that describe alternative socioeconomic developments that contribute different land use and carbon emissions (Pörtner 2002; Meinhausen et al. 2020; Gidden et al. 2019). SSP 126 is a climate scenario that assumes that global warming is limited to below 2°C, while SSP 370 is a climate scenario that assumes that global warming surpasses 2°C (Meinhausen et al. 2020). These climate datasets were chosen because they are heavily researched and depict the future global climate at a high resolution. We calculated the difference between historical and future WorldClim temperature seasonality and precipitation seasonality experienced by each species across their breeding distribution. Future greenness seasonality projections of the same resolution and for the same global climate model were not available.

All analyses were conducted in the R programming language (R Core Team 2021).

## 3 Results

**FIGURE 1:**
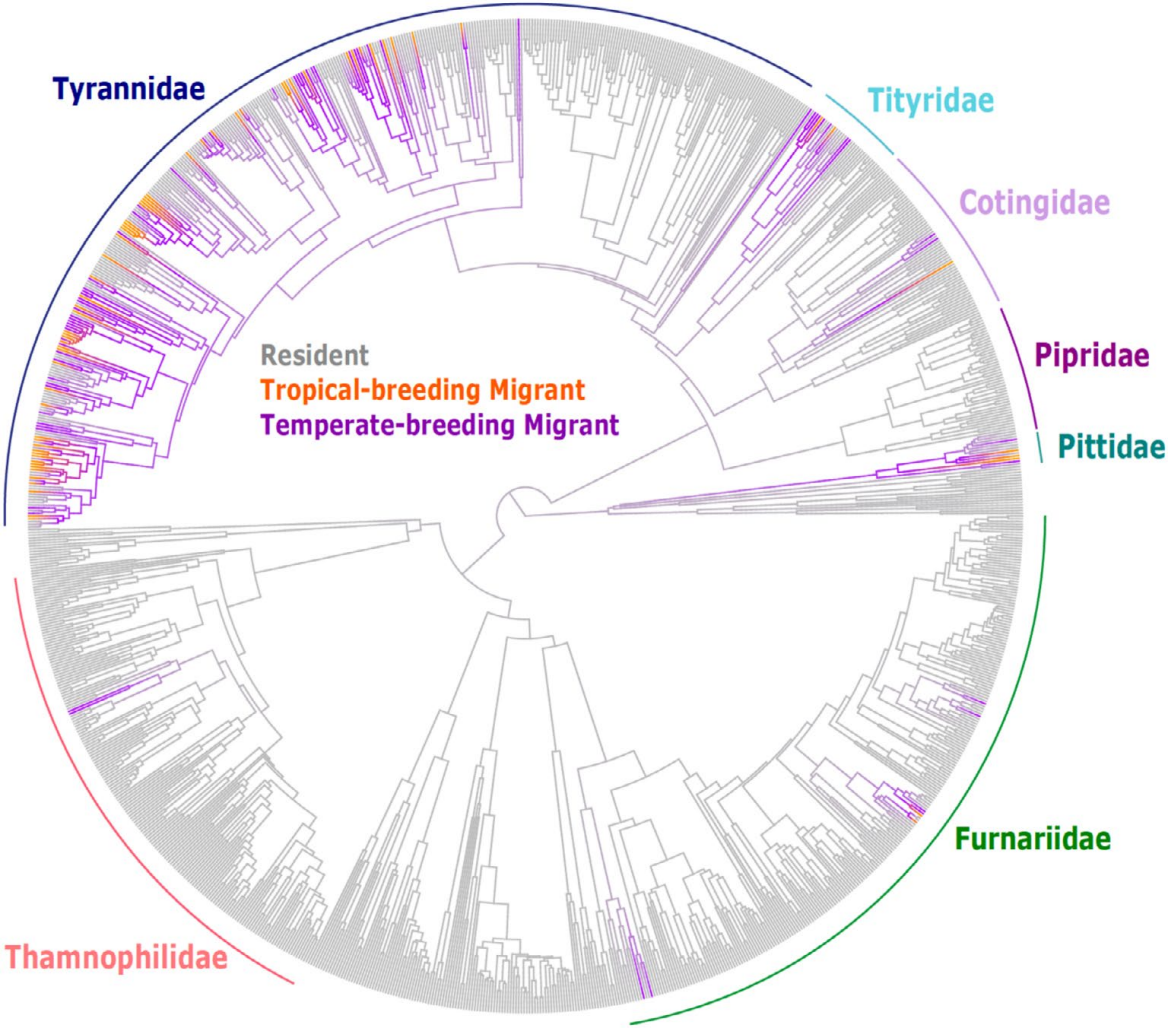
Ancestral state reconstruction of migration on the suboscine phylogeny. Migratory behavior of every suboscine species is characterized as resident (gray), tropical‐breeding migrant (orange), or temperate‐breeding migrant (purple). Colored branches indicate migratory species. The transition from resident to migratory is marked by the point at which the branches transition from gray to purple or orange. The ancestral state of migration is resident, and this map depicts 37 independent origins of migration and 64 losses.

In an ancestral state reconstruction with partial migrants classified as migratory, we identified 37 independent gains and 64 losses of migration on the suboscine phylogeny (Figure 1). This high number of gains and losses suggests that there are enough independent emergences of the behavior to provide power to test the causes of those shifts. We also found evidence that migratory behavior shows a strong phylogenetic signal (Blomberg's K = 0.16, Pagel's lambda = 0.47), which suggests that controlling evolutionary history is important in the comparative analysis of this trait.

In the following results from comparative tests, we focus on those using the latitudinal coding of migration (temperate‐breeding versus tropical‐breeding). Results from the distance‐based coding were similar and are presented in Figures S1 and S2.

### 3.1 Climate Seasonality and Migration

**FIGURE 2:**
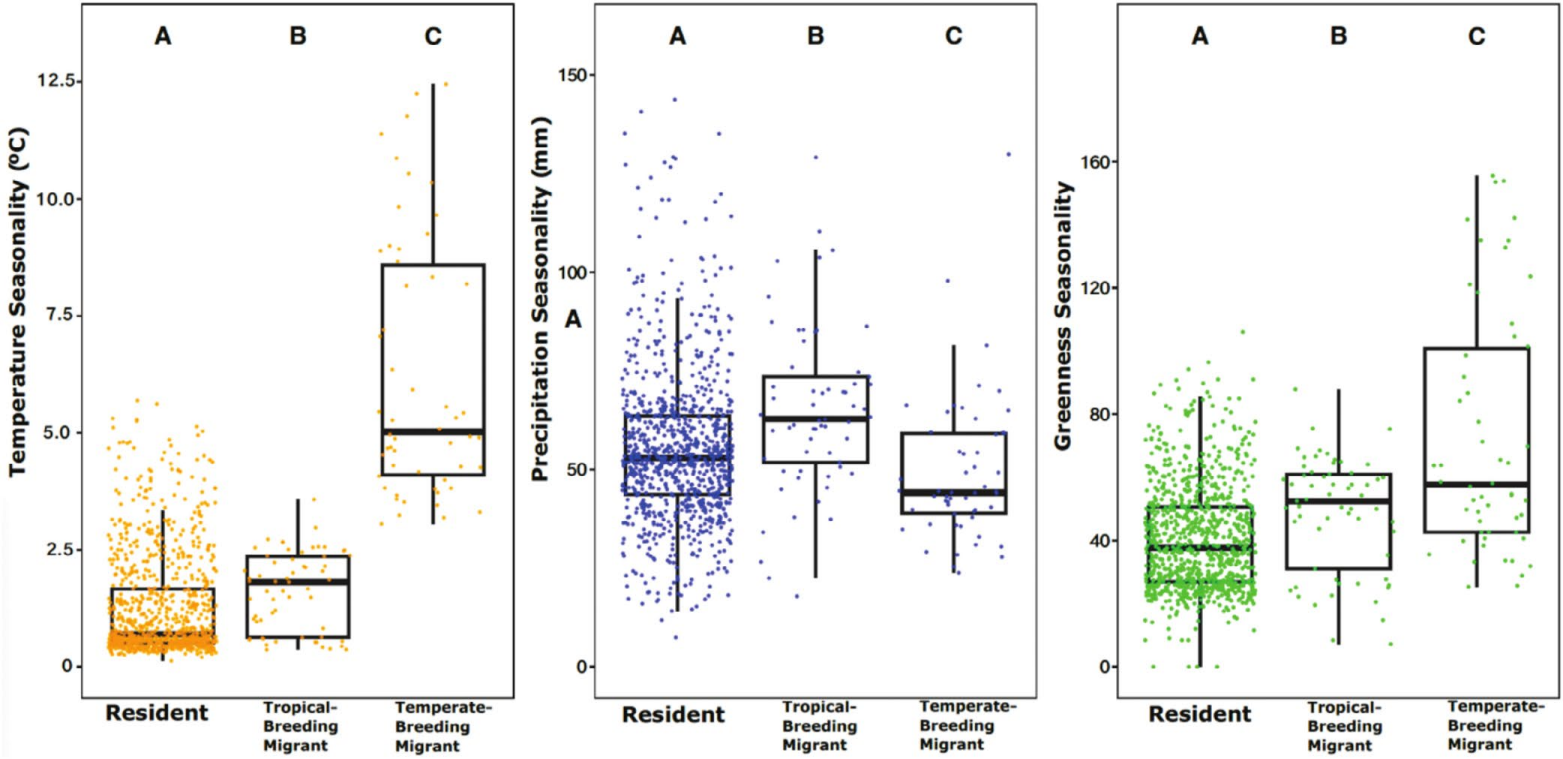
Temperature seasonality, precipitation seasonality, and greenness seasonality vary depending on migratory behavior. Left: Birds with different migratory strategies live in climates with different degrees of temperature seasonality. Temperature seasonality is significantly associated with migration, regardless of migratory behavior (resident *p* = 2.00E‐16, tropical‐breeding migrant *p* = 3.12E‐02, temperate‐breeding migrant *p* = 2.00E‐16). Average temperature seasonality, in degrees Celsius, of breeding‐resident range is plotted on the vertical axis. Middle: Birds with different migratory strategies live in climates with statistically different degrees of precipitation seasonality. Precipitation seasonality is significantly associated with migration strategy, regardless of their behavior (resident *p* = 2.00E‐16, tropical‐breeding migrants *p* = 1.55E04, temperate‐breeding migrants *p* = 0.0259). Average precipitation seasonality, in millimeters, of each bird's breeding‐resident range is plotted on the vertical axis. Right: Birds with different migratory strategies live in climates with statistically different degrees of greenness seasonality. Greenness seasonality is significantly associated with migration, regardless of migratory behavior (resident *p* = 2.00E‐16, tropical‐breeding migrant *p* = 1.40E‐03, temperate‐breeding migrant *p* = 2.00E‐16). Average greenness seasonality, unitless, of breeding‐resident ranges is plotted on the vertical axis.

We found that residents, tropical breeders, and temperate breeders each live in areas with different degrees of temperature seasonality (TS), precipitation seasonality (PS), and greenness seasonality (GS), based on nonoverlapping 95% confidence intervals of phylogenetic ANOVAs (Figure 2). In particular, we found that temperate‐breeding migrants live in climates with the highest degree of TS (mean = 6.24°C), as compared to residents (mean = 1.26°C) and tropical‐breeding migrants (mean = 1.61°C) and in climates with the greatest degree of GS (mean = 72.84), as compared to residents (mean = 40.51) and tropical‐breeding migrants (mean = 48.39) (Figure 2). In an ANOVA, TS showed a significant positive relationship with migration in residents (*p* = 2.00E‐16), tropical‐breeding migrants (*p* = 3.12E‐02), and temperate‐breeding migrants (*p* = 2.00E‐16), while GS showed a significant relationship with migration in residents (*p* = 2.00E‐16), tropical‐breeding migrants (*p* = 1.40E‐03), and temperate‐breeding migrants (*p* = 2.00E‐16). In contrast, tropical‐breeding migrants live in climates with the greatest degree of PS (mean = 65.00 mm), as compared to residents (mean = 55.20 mm) and temperate‐breeding migrants (mean = 49.10 mm; Figure 2). In an ANOVA, PS showed a significant relationship with migration in residents (*p* = 2.00E‐16), tropical‐breeding migrants (*p* = 1.55E‐04), and temperate‐breeding migrants (*p* = 0.026). Other WorldClim variables aside from PS and TS also showed correlations with migratory behavior, which are presented in Appendix S1.

### 3.2 Causal Links Between Climate and Migration

**FIGURE 3:**
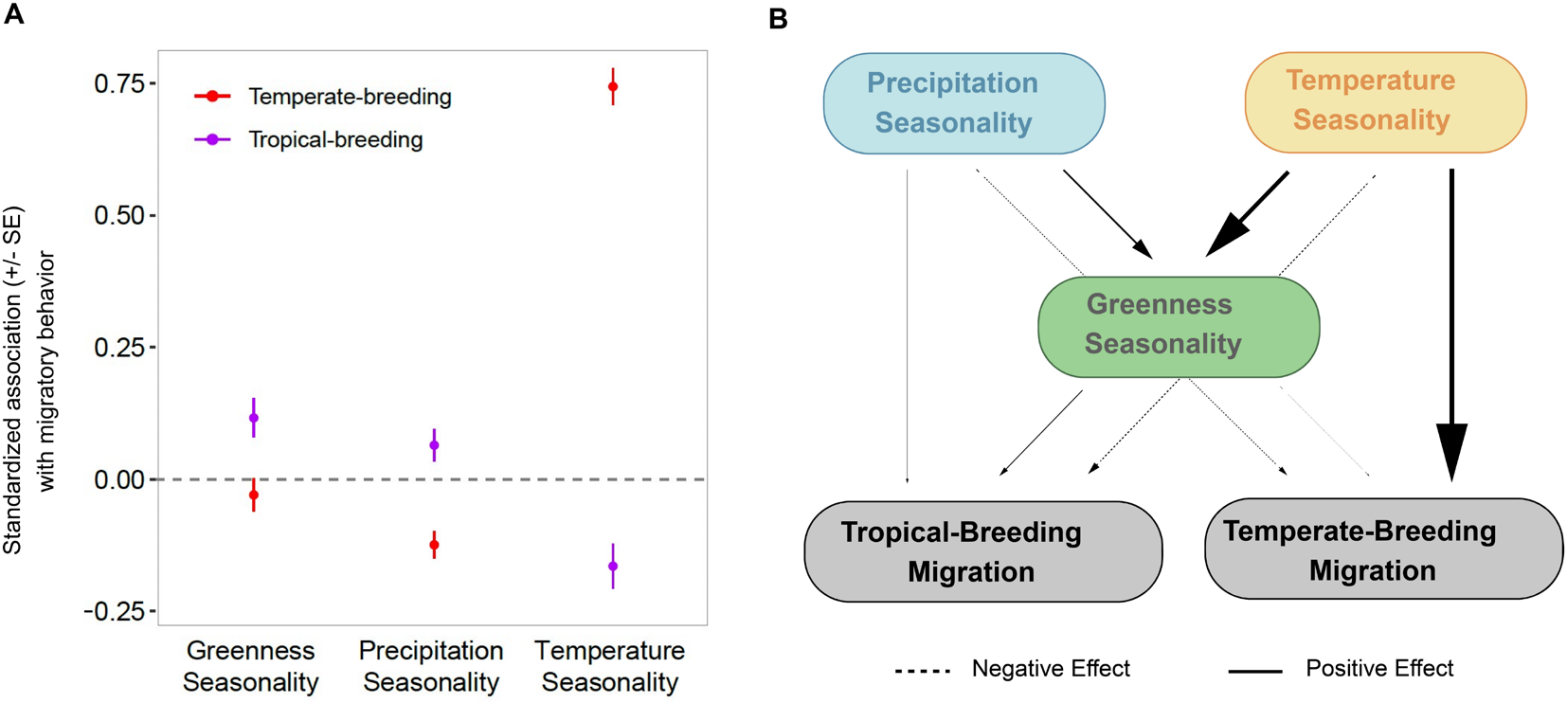
Phylogenetic path analysis. (A) Plot depicting the standardized associations (effect magnitudes) of greenness seasonality, precipitation seasonality, and temperature seasonality with respect to migrants breeding in tropical and temperate climate zones. Of the three climate variables, temperature seasonality has an overwhelmingly strong association (0.74) with migration in temperate‐breeding birds. Precipitation seasonality and greenness seasonality have moderate (−0.12) and weak (−0.03) negative relationships with migration in temperate‐breeding migrants, respectively. All three climate variables show weak to moderate relationships with migration in tropical‐breeding birds (temperature seasonality: −0.16, precipitation seasonality: 0.06, greenness seasonality: 0.12). (B) Phylogenetic path analysis illustrating the interactions between climate variables and different migratory behaviors. Thicker lines represent stronger associations. The association between temperature seasonality and migration in temperate‐breeding birds is the strongest. The association between greenness seasonality and migration in temperate‐breeding birds is the weakest.

The PPA identified strong relationships between TS and temperate‐breeding migrants (0.74) and between TS and GS (0.63; Figure 3B). The PPA identified moderate relationships between PS and GS (0.29), TS and tropical‐breeding birds (−0.16), PS and temperate‐breeding birds (−0.12), and GS and tropical‐breeding birds (0.12). Weak links were present between PS and tropical‐breeding birds (0.06) and GS and temperate‐breeding birds (−0.03). Overall, migratory behavior in temperate breeders had a causal chain dominated by a direct link with TS, whereas in tropical breeders, the causal chain was complex with moderate direct impacts from both PS and TS and indirect effects mediated by GS (Figure 3A).

### 3.3 Geography of Seasonality

**FIGURE 4:**
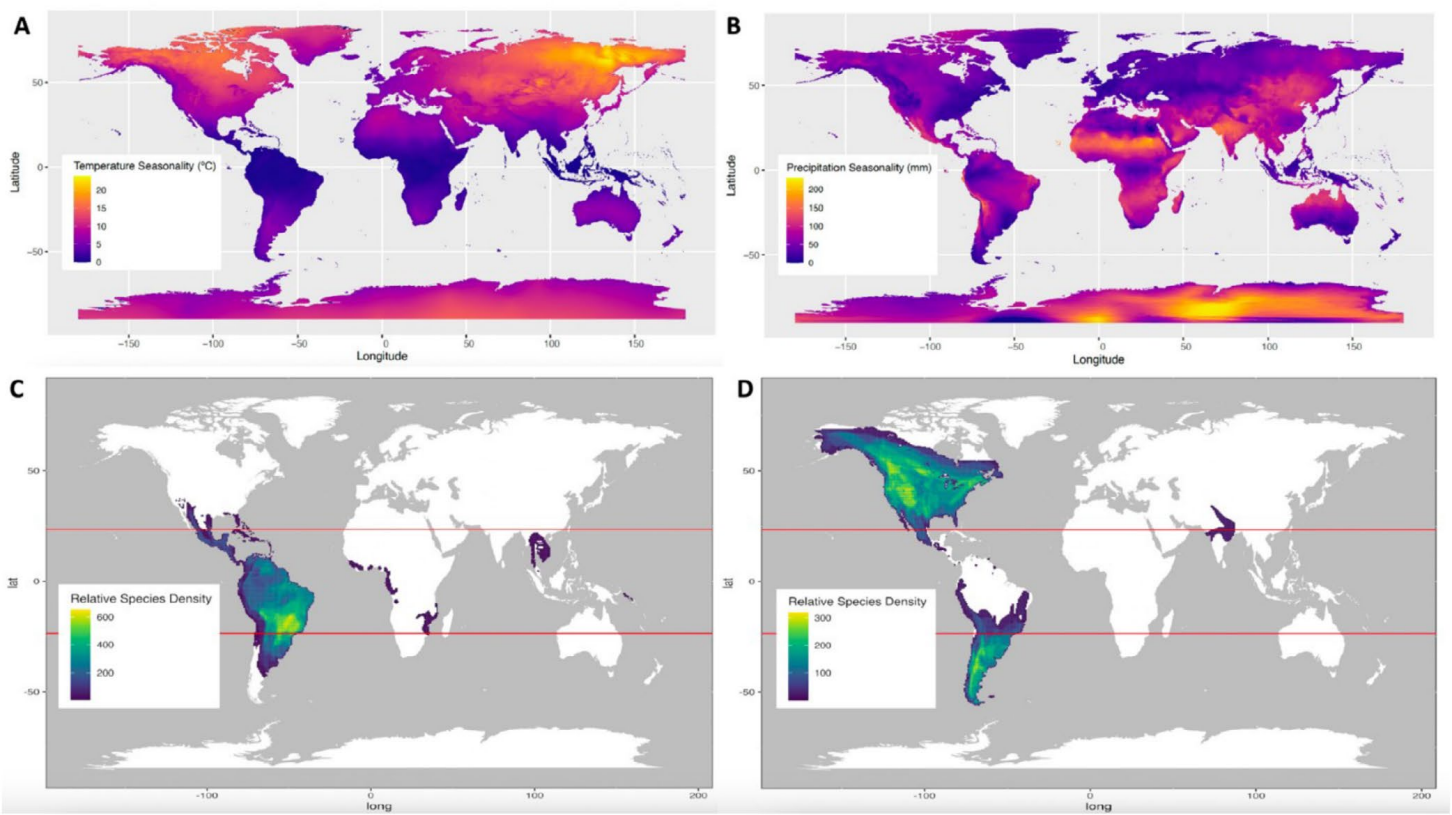
Species density plots for tropical‐ and temperate‐breeding migrants with maps of temperature and precipitation seasonality. (A) Temperature seasonality, in degrees Celsius, is plotted over the world map. (B) Precipitation seasonality, in millimeters, is plotted over the world map. (C) Species density of tropical‐breeding birds. (D) Species density of temperate‐breeding birds. In C and D, the red lines represent the Tropic of Cancer (23° 26′ 22″ North) and Tropic of Capricorn (23° 26′ 22″ South). Bound between the tropics is the tropical climate breeding zone. The northern temperate zone begins above the top red line, or the Tropic of Cancer. Temperate‐breeding birds are depicted to live in areas with a broader range of temperature seasonality, and, on average, higher temperature seasonality than tropical‐breeding birds. The area with the highest density of tropical‐breeding species, east‐central South America, has a relatively higher degree of precipitation seasonality than those areas where temperate‐breeding migrant species are most abundant.

Maps of TS and PS and species density plots for tropical‐ and temperate‐breeding migrants reveal differences in the climates that birds inhabit depending on their migratory strategy (Figure 4). Temperate‐breeding migrants have breeding ranges concentrated in the highest latitudes and in mountainous areas of western North America and southern South America and experience the highest TS (Figure 4A,D). Tropical‐breeding migrants in the Americas are most concentrated in the “Dry Triangle” of eastern South America and north‐central South America, an area with high PS (Figure 4B,C). These results corroborate those from the phylogenetic ANOVA (Figure 2), showing that PS is relatively low where both tropical‐ and temperate‐breeding migratory birds reside, is higher where tropical‐breeding birds are concentrated, and is variable in areas with many temperate breeders but much lower than TS in those zones.

### 3.4 Taxonomic Variation in Climate‐Migration Associations

**FIGURE 5:**
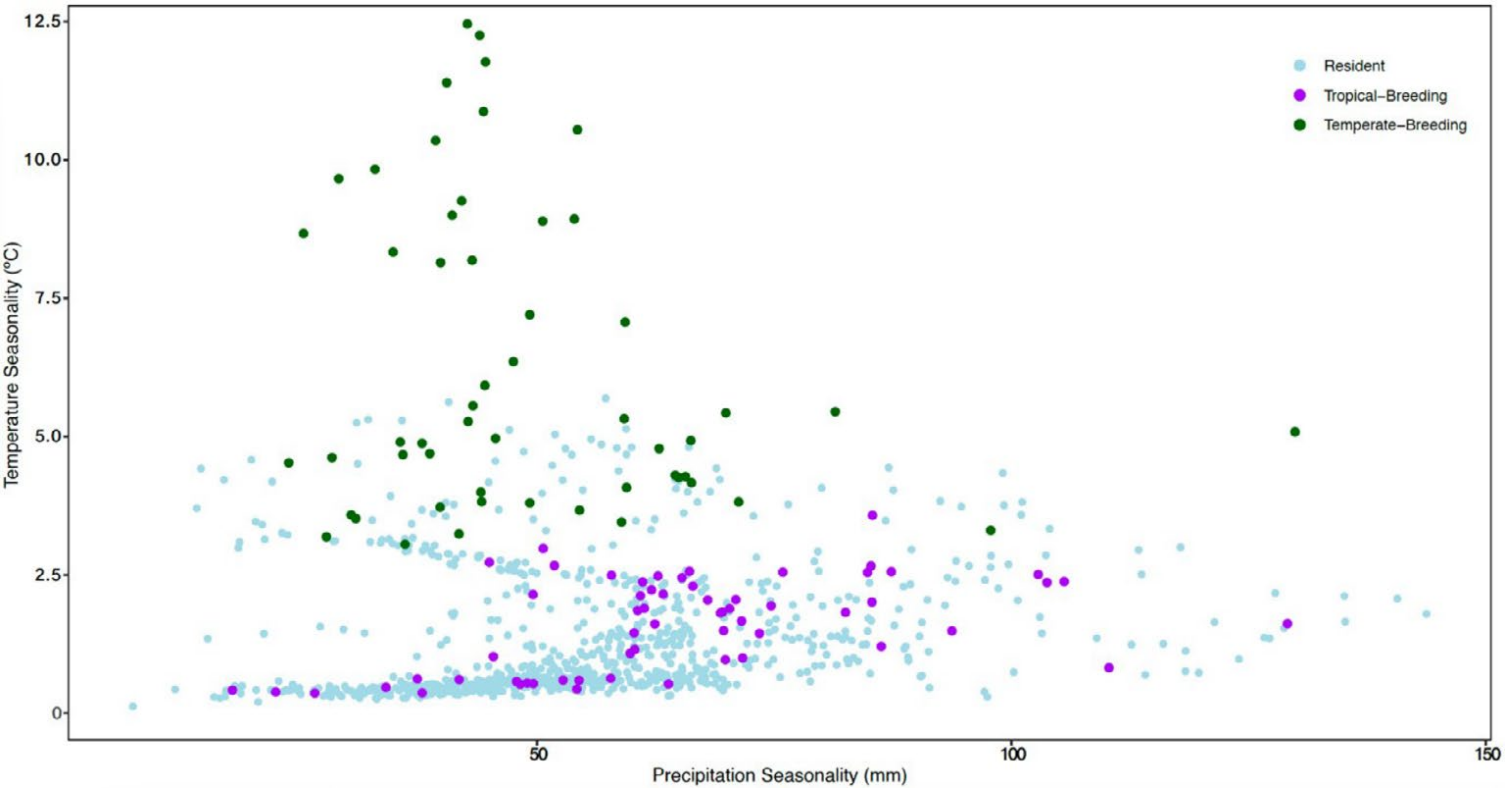
Temperate‐ and tropical‐breeding migrants and resident bird species are concentrated in different areas when plotted on axes representing both temperature and precipitation seasonality. Temperate‐breeding migrants live in climates with high temperature seasonality but low precipitation seasonality, whereas tropical‐breeding migrants live in climates with low temperature seasonality and varying but generally higher precipitation seasonality. There is extensive overlap between residents and tropical‐breeding migrants, but areas of lower precipitation seasonality are enriched for residents relative to migrants.

We found that suboscine species experience annual fluctuations in precipitation across their breeding distribution that range from 17.88 to 143.74 mm and annual fluctuations in temperature that range from 0.36°C to 12.45°C (Figure 5). Climates with particularly low TS (seasonality less than 1°C) contained both residents and tropical‐breeding migrants, but the species that experiences the lowest degree of TS (0.363°C) is the tropical‐breeding resident *Elaenia brachyptera* of the Andean foothills. We did not detect any general taxonomic trends or dominant genera in these stable climates. In regions of high TS, where seasonality is greater than 7.5°C, only temperate‐breeding birds occurred. The long‐distance, temperate‐breeding migrant *Empidonax flaviventris* experiences the highest TS in its breeding grounds of 12.45°C. Of the 17 species living in high‐TS climates, eight belong to the genus *Empidonax*.

Climates with low PS, where seasonality is less than 50 mm, were home to residents and both tropical‐ and temperate‐breeding birds. The tropical‐breeding *Pitta anerythra*, one of the relatively few Eastern Hemisphere suboscines, experiences the lowest PS (17.88 mm). Of the 49 temperate‐ and tropical‐breeding birds living in areas with low PS, 10 belong to the rather large genus *Empidonax*. Climates with high PS (where seasonality is over 100 mm) contain tropical‐ and temperate‐breeding birds. Unlike in climates with high TS, several resident species exist in areas with high PS. Interestingly, the species that experiences the highest degree of PS (143.74 mm) is *Ochthoeca salvini* , a resident species or nearly so (Farnsworth and Langham 2022). The only temperate‐breeding bird in high PS climates is *Pitta brachyura* , which occurs in areas with an average PS of 129.88 mm and a moderate TS of 5.08°C. There were no clear taxonomic patterns or dominant genera of birds in climates with high PS.

### 3.5 The Influence of Climate Change on Breeding Range Climate

**FIGURE 6:**
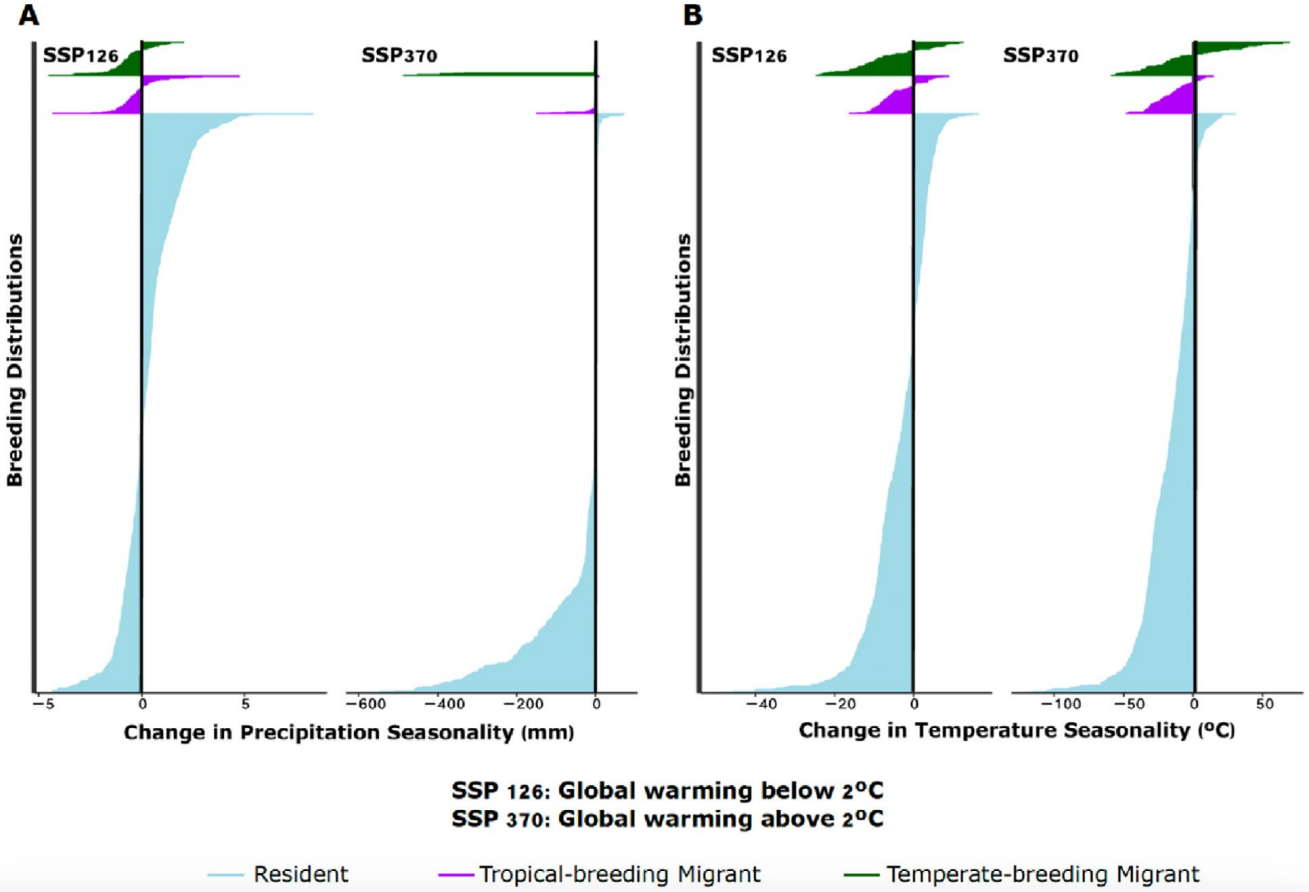
(A) The difference was calculated between historical and future temperature seasonality values for two different Shared Socioeconomic Pathways, SSP 126 and SSP 370. Change in temperature seasonality, in degrees Celsius, is plotted on the horizontal axis, and each species is plotted on the vertical axis. On average, temperature seasonality is expected to decline across the breeding ranges of suboscine species, regardless of migratory strategy or Shared Socioeconomic Pathway. (B) The difference was calculated between historical and future precipitation seasonality values for two different Shared Socioeconomic Pathways, SSP 126 and SSP 370. Change in precipitation seasonality, in millimeters, is plotted on the horizontal axis, and each species is plotted on the vertical axis. Under SSP 126, residents will experience a slight increase in precipitation seasonality across their breeding ranges, while tropical‐breeding and temperate‐breeding migrants will experience a slight decrease in precipitation seasonality. Under SSP 370, residents, tropical‐breeding migrants, and temperate‐breeding migrants will experience moderate to large decreases in precipitation seasonality across their breeding ranges. Color to the left of the *x* = 0 indicates a decrease in climate seasonality, while color to the right of *x* = 0 indicates an increase in climate seasonality.

When we evaluated the changes in TS and PS under two different future climate scenarios, we found that while TS, on average, is expected to decrease across breeding distributions regardless of migratory strategy or climate scenario (Figure 6A), PS is less predictable (Figure 6B). Under SSP 126, TS is expected to decrease by an average of 3.63°C, 4.35°C, and 7.30°C across the breeding distributions of residents, tropical‐breeding migrants, and temperate‐breeding migrants, respectively (Table S2).

Under SSP 370, TS is expected to decrease by an average of 17.47°C, 16.95°C, and 5.61°C across the breeding distributions of residents, tropical‐breeding migrants, and temperate‐breeding migrants, respectively (Table S2).

PS across the breeding ranges of suboscine birds may increase or decrease depending on both the migratory strategy and future climate scenario. Under SSP 126, residents are expected to experience an average increase in PS by 0.29 mm across their breeding distributions, while tropical‐breeding migrants and temperate‐breeding migrants will experience, on average, a small decrease in PS by 0.25 and 0.71 mm, respectively (Table S2). Under SSP 370, PS will decrease to a much greater degree for all suboscine species, regardless of migratory strategy (Figure 6B). On average, PS will decrease by 39.79, 8.50, and 39.54 mm across the breeding ranges of residents, tropical‐breeding migrants, and temperate‐breeding migrants, respectively (Table S2). Based on these results, it is evident that the migratory behavior of suboscines vulnerable to PS is expected to differ based on the severity of climate change. Under a less severe scenario, while residents may evolve migration, current migratory species are expected to respond accordingly. Under more severe climate change, a decrease in PS is expected to cause a trend toward shorter‐distance migratory movements in suboscines, regardless of their current behavior.

## 4 Discussion

We found that the evolution of long‐distance, temperate‐tropical migration in suboscine birds is strongly associated with temperature seasonality on the breeding grounds. This supports prior findings that long‐distance migratory movements are a strategy to avoid the seasonal resource depletion that occurs within breeding territories during the harsh winter months (Bell 2000; Bell 2011; Alerstam et al. 2003; Cresswell et al. 2011; Winger et al. 2019). Temperature is recognized as a primary driver of natural history and behavior, with strong effects on population growth rates (Neate‐Clegg et al. 2021), distribution (Avalos and Hernández 2015; Somveille et al. 2015; Tellería et al. 2016; Rushing et al. 2020; Carrera et al. 2022), and the timing of migration (Zaifman et al. 2017; Koleček et al. 2020). Changes in temperature have been shown to be a proximate mediating cue in many of these behaviors, including long‐distance migration (Klinner and Schmaljohann 2020), which corroborates our finding that longer‐distance migrations are tied to temperature seasonality.

However, there is no clear consensus on the mechanism by which temperature influences migratory behavior. Although increasing spring temperatures have been directly correlated to the timing and rate of migration in several long‐distance migratory species, stronger correlations have been found between first budburst in the plants upon which these species rely and their spring arrival dates (Marra et al. 2005). This suggests that changes in temperature are influencing bird phenology indirectly via changes in plant phenology (Reid et al. 2022). Our path analysis results, however, seem to indicate that temperature seasonality is directly influencing migratory behavior in temperate‐breeding migrants rather than indirectly through vegetation greenness seasonality (Figure 3B), potentially implicating physiological tolerance. Clearly, more work is needed to understand the mechanism by which temperature seasonality influences the evolution of migration.

In contrast, we found that short‐distance, intratropical migration is influenced by a broader variety of factors. Precipitation seasonality is an especially important predictor of intratropical movements. This may not be surprising given that movements in several well‐studied intratropical migrants appear to track rainfall (Jahn et al. 2010; MacPherson et al. 2018) and populations of some tropical birds are associated with fluctuations in seasonal rainfall (Brawn et al. 2016; Liang et al. 2021; Brlík et al. 2022). Further, rainfall, like temperature, influences the behavior, life history, physiology, and morphology of birds (McGowan et al. 2004; Skagen and Adams 2012; Öberg et al. 2014; Mainwaring et al. 2021) and impacts both the timing and location of breeding (Aranzamendi et al. 2019; Fogarty et al. 2020). The effect of precipitation seasonality on intratropical migration likely co‐varies also with greenness seasonality. The seasonal movements of animals have long been associated with variation in ecological productivity (Dingle 2014; Milner‐Gulland et al. 2011; Buchanan et al. 2016). Rainfall has a substantial influence on plant phenology (Both et al. 2010; Currier and Sala 2022) and the abundance of arthropods (Pinheiro et al. 2002), but shifting rainfall dynamics brought on by climate change are influencing the timing, quality, and quantity of floral resources upon which both birds and their invertebrate food sources rely (Geissler et al. 2023; Thuma et al. 2023). Our results suggest that there are important indirect effects of temperature and precipitation on migratory strategy via their influence on greenness seasonality, supporting the idea that primary productivity is an important driver of the migration dynamics of birds (Studds and Marra 2011; Illán et al. 2014; Koshelev et al. 2021). Our confirmation of the importance of precipitation seasonality on the evolution of migration emphasizes the important role of wet‐dry cycles on bird movements in tropical regions.

Due to the highly complex effects of precipitation seasonality, we expect the responses of intratropical migrants to be species‐specific and context‐dependent. Santillán et al. (2018) concluded that birds at different elevations were impacted by climate uniquely due to their different physiological constraints under their respective climatic niches. Further, the influence of climatic seasonality is less dependent on a bird's migratory behavior and more on geography (Both et al. 2010; de la Fuente et al. 2023) or dietary guild (MacPherson et al. 2018). Further work characterizing the migratory strategies of intratropical migrants and linking them to environmental and climatic variation is urgently needed to understand the complexities of species responses to past and future change. Interestingly, all suboscines in regions of high temperature seasonality are migratory, while regions of high precipitation seasonality exhibit a mixture of migratory and resident species. This suggests that, in this group at least, migration is an obligate response to temperature seasonality, while migration due to precipitation seasonality is facultative. Although resident birds from other passerine groups occur in areas experiencing high fluctuations in temperature, most high‐latitude suboscines are flycatchers (Tyrannidae). These are sallying insectivores that rely on insect activity for foraging and likely have no alternate strategies for surviving cold winters when insects are dormant (Sherry et al. 2020). In these species, therefore, migration serves as the best evolutionary strategy for avoiding the cold and barren winter conditions lacking insects (Lack 1968; Chesser and Levey 1998; Bell 2000; Alerstam et al. 2003; Cresswell et al. 2011). This is consistent with the idea that, for birds with specialized diets, dietary guild may be a key predictor to understanding how migration evolves (Chesser and Levey 1998; Benson and Winker 2001). It also supports theoretical frameworks positing that long‐distance migration allows birds to experience the benefits associated with maintaining a consistent breeding territory while avoiding seasonal resource droughts (Winger et al. 2019), thereby maximizing their overall fitness.

In contrast, suboscines breeding in areas of high precipitation seasonality are more taxonomically diverse, belonging to 10 families (Tyannidae, Pittidae, Rhinocryptidae, Grallariidae, Melanopareiidae, Furnariidae, Eurylaimidae, Thamnophilidae, Cotingidae, and Tityridae). They have a wider array of diets (Correa et al. 1990; Sillett et al. 1997; Zubkova and Korzun 2014; Buainain et al. 2015; Greeney and Vargas‐Jiménez 2017; Cataudela and Palacio 2021) and may have more options for coping with resource depletion in dry seasons, without undertaking risky migrations. For example, opportunistic and innovative foraging strategies give residents the ability to exploit dry environments by rapidly locating and obtaining resources, which improve their chances of survival during food shortages, without migrating (Sol et al. 2005). Additionally, many birds have evolved behavioral and physiological adaptations to maximize their fitness in dry conditions, which range from avoiding strenuous activity during the heat of the day to reduce their heat load and water loss (Davies 1982) and the nonparticipation in aggressive or competitive interactions with other individuals (Thomas 1981), to adaptive heterothermy (Smit et al. 2013) and a tight regulation of metabolic pathways (Ribeiro et al. 2019). Notably, however, all migrant species in areas of high precipitation seasonality again belong to the Tyrannidae, which may be more dependent on insect activity than other suboscines. Representatives of the other families are all residents, indicating that there are other means for surviving dry conditions for these species. Regardless, avian responses to changes in climate, and in particular to changes in precipitation, are likely more granular than might be explained by breeding zone, migration strategy, location, or dietary guild alone. The evolution of migration in many cases may therefore be driven by combinations of species‐specific factors.

To explore the sensitivity of our results, we coded migration using two methods (1) a categorical measure based on whether a species bred in temperate or tropical latitudes and (2) a categorical measure of migration distance. The Tropics of Cancer and Capricorn provide clear thresholds for grouping taxa for the latitudinal coding, while the migration distance measurement does not. Additionally, the distance‐based coding method groups species that experience drastically different climates. Therefore, our analyses primarily examined migrants coded as either temperate‐tropical or intratropical. However, the latitude‐based method has some disadvantages. The range centroid might place species with wide latitudinal distributions in the tropics even if a majority of their range is in the temperate zone. We confirmed this was not an issue here, however, by manually examining the ranges of each species. Using a latitudinal categorization to test the effect of climate may also be somewhat circular because climate changes with latitude. However, we found highly concordant results using the two methods of coding migratory behavior (Figures S1 and S2), indicating that our results are robust to the different methodologies. The phylogenetic ANOVAs demonstrated all the same relationships between each climatic variable and migratory behavior, with only a few relationships showing weaker associations when using distance‐based coding. Specifically, long‐distance migrants did not differ from residents or short‐distance migrants with the distance‐based coding, whereas all three groups (residents, temperate‐breeding migrants, tropical‐breeding migrants) differed using the latitudinal coding. This suggests that it is primarily the short‐distance migrants that are impacted by precipitation seasonality, a result that does not conflict with our major conclusions and only adds nuance to the dynamics evident in the results from the latitudinal coding. Key results, like a strong link between long‐distance migrants and temperature seasonality versus the complex causal links to shorter‐distance migrants in the PPA (Figure S2) were consistent with those from the latitudinal coding. We acknowledge that migratory strategies are diverse, and any categorization might conflate disparate movements (see Figure S3). Future work should focus on improved approaches to categorizing diverse behaviors into biologically meaningful groups.

The different environmental drivers of migration in temperate‐ versus tropical‐breeding suboscines implies that these species may have different responses to future climatic change. Although migration in birds is generally plastic and evolutionarily labile (Zink 2002, 2011), long‐distance migration often involves highly specialized morphologies (Klaassen et al. 2012; Handby et al. 2022) and may require a constrained set of genetic variants at key genes (Gu et al. 2021), which could make it less flexible than shorter‐distance migrations. Because of their more varied environmental drivers, migration in tropical systems could be more plastic in the face of changing climatic conditions. Perhaps tropical‐breeding migrants might be able to switch more readily between migratory and resident strategies, given the high rate of loss of migration across the suboscine phylogeny and the large number of resident species already present in these areas.

Forecasting changes in migratory behavior is critical to protecting birds and the natural phenomenon that is avian migration. Our analysis of breeding range climate under two future climate change scenarios revealed that temperature seasonality is expected to decline for all suboscine species, regardless of migratory strategy. Greater temperature stability across the breeding distribution will likely favor a decrease in the propensity to migrate and, on a longer evolutionary timescale, could promote the loss of migration in temperate‐breeding migratory birds (Heldbjerg and Fox 2008).

Interestingly, changes in precipitation seasonality are much less uniform. Under the more optimistic climate scenario, SSP 126, resident species will experience an increase in precipitation seasonality across their breeding distribution, while temperate‐ and tropical‐breeding migratory species will experience a slight decrease. Further, under SSP 370, a more severe climate scenario, precipitation seasonality is expected to decrease substantially across the breeding ranges of all suboscine species, regardless of migratory strategy. The projected increase in precipitation seasonality that will be experienced by resident species under SSP 126 may favor an increase in migratory behavior, with some current resident, tropical‐breeding species shifting to at least short‐distance, intratropical migration (Williams and Middleton 2008; Sekercioglu 2010; Sekercioglu et al. 2012), assuming that they can evolve this migration strategy. In contrast, large decreases in precipitation seasonality could favor an overall decrease in migratory behavior. The divergent response of precipitation seasonality to different climate scenarios corroborates our conclusion that shorter‐distance, intratropical movements are complex, making responses to future climate change challenging to predict.

Overall, it is evident that we expect to see changes in migratory behavior in response to climate change. The trajectories that migratory avifauna take in response to future climatic change will shape not only their outcomes, but also overall community structure (Carey 2009), ecosystem function (Morante‐Filho and Faria 2020; Wilson et al. 2022; Zhang et al. 2023), and ecosystem services (Jourdain et al. 2007; Pejchar et al. 2018; Dvořáková et al. 2023). While there are many unanswered questions regarding what thresholds of climatic change will trigger a change in migratory behavior and on what timescales these changes may occur, this work provides a foundation for our understanding of how climate seasonality influences migration in a rapidly changing world.

## Author Contributions

**Emily X. Johns:** conceptualization (equal), data curation (equal), formal analysis (equal), investigation (equal), methodology (equal), project administration (equal), software (equal), validation (equal), visualization (equal), writing – original draft (lead), writing – review and editing (lead). **Oscar Johnson:** conceptualization (equal), data curation (equal), formal analysis (equal), investigation (equal), methodology (equal), project administration (equal), resources (equal), software (equal), supervision (equal), validation (equal), visualization (equal), writing – review and editing (equal). **Michael G. Harvey:** conceptualization (equal), funding acquisition (equal), methodology (equal), project administration (equal), supervision (lead), validation (equal), writing – review and editing (equal).

## Conflicts of Interest

The authors declare no conflicts of interest.

## Supporting information


Appendix S1.

## Data Availability

All code and data are available on GitHub. After acceptance, all code will be archived at Zenodo and data will be archived at Dryad. https://doi.org/10.5061/dryad.xgxd254s4.
