# Correction to “Migration Evolves in Response to Distinct Regimes of Climate Seasonality in Tropical Versus Temperate‐Breeding Suboscine Birds”

**DOI:** 10.1002/ece3.71950

**Published:** 2025-08-07

E. X. Johns, O. Johnson, M. G Harvey, “Migration evolves in response to distinct regimes of climate seasonality in tropical versus temperate‐breeding suboscine birds,” *Ecology and Evolution* 15, no. 7 (2025): e71745, https://doi.org/10.1002/ece3.71745.

The authors would like to note the following corrections in the published article.

1. The Acknowledgments section has been updated to include additional information and to correct Terry R. Chesser's name.

The correct Acknowledgements should read as follows:

