# Supplementary material for: Migration Evolves in Response to Distinct Regimes of Climate Seasonality in Tropical Versus Temperate‐Breeding Suboscine Birds
Source: Ecol Evol. 2025 Jul 9;15(7):e71745. doi: 10.1002/ece3.71745 (PMC12240598; doi:10.1002/ece3.71745)
Supplement: Supplementary file 1 — Appendix S1. [file ECE3-15-e71745-s001.docx]

**Supporting Information**

**Appendix S1.** To evaluate potential causal factors responsible for the evolution of migration, we performed phylogenetic generalized least squares (PGLS) regressions on migration status (resident, temperate-breeding, tropical-breeding) versus each of the 18 WorldClim Bioclimatic variables and vegetation greenness seasonality.

In addition, we examined regression models with all predictors jointly and then iteratively removed one of the 19 bioclimatic variables (a “leave-one-out" approach to evaluating predictors), and calculated the Akaike Information Criterion (AIC) values for each model. The difference was calculated between the AIC values of the complete model and each of the partial models, with a difference (ΔAIC) of greater than or equal to the absolute value of 2 treated as important.

Eleven of the 19 bioclimatic variables were significantly correlated with migratory behavior in our AIC analysis. The variables that produced a ΔAIC of greater than or equal to the absolute value of 2 were BIO1 (annual mean temperature; -2.075), BIO2 (mean diurnal range; 6.721), BIO3 (isothermality; 29.219), BIO4 (temperature seasonality; 9.236), BIO5 (max temperature of the warmest month; 4.538),

BIO6 (min temperature of the warmest month; 2.846), BIO8 (mean temperature of the wettest quarter; 7.734), BIO10 (mean temperature of the warmest quarter; 7.685), BIO11 (mean temperature of the coldest quarter; 7.608), BIO12 (annual precipitation; -2.006), and BIO16 (precipitation of the wettest quarter; -2.066).

Understanding the mechanism by which climate influences migration was challenging due to the strong covariation between variables. While temperature-related variables generally had a stronger influence on migratory behavior, many temperature and precipitation variables showed high covariation. Based on previous work that has focused on understanding the influence of climate on the evolution of migration, we proceeded with BIO4 (temperature seasonality) and BIO15 (precipitation seasonality) in the following analyses. We included greenness seasonality in these models to evaluate the role of vegetation as an intermediate between climate and migratory behavior.

**Table S1.** Results of AIC analysis of all WorldClim bioclimatic variables and migration. We examined 19 PGLS regression models with migration status as the dependent variable, and the bioclimatic variables as the independent variables, in which we iteratively removed one of the climatic variables in each model. The difference was calculated between the AIC values of the complete model and each of the partial models, with a difference (ΔAIC) of greater than or equal to the absolute value of 2 treated as important. BIO7 Temperature Annual Range, a composite variable of BIO5 Max Temperature of Warmest Month and BIO6 Min Temperature of Coldest Month, was not included in the analysis due to its high interrelatedness with the other two. BIO4 Temperature Seasonality and BIO15 Precipitation Seasonality, which were focal to this study, are defined as the standard deviation of temperature multiplied by 100 and the coefficient of variation of monthly precipitation, respectively.


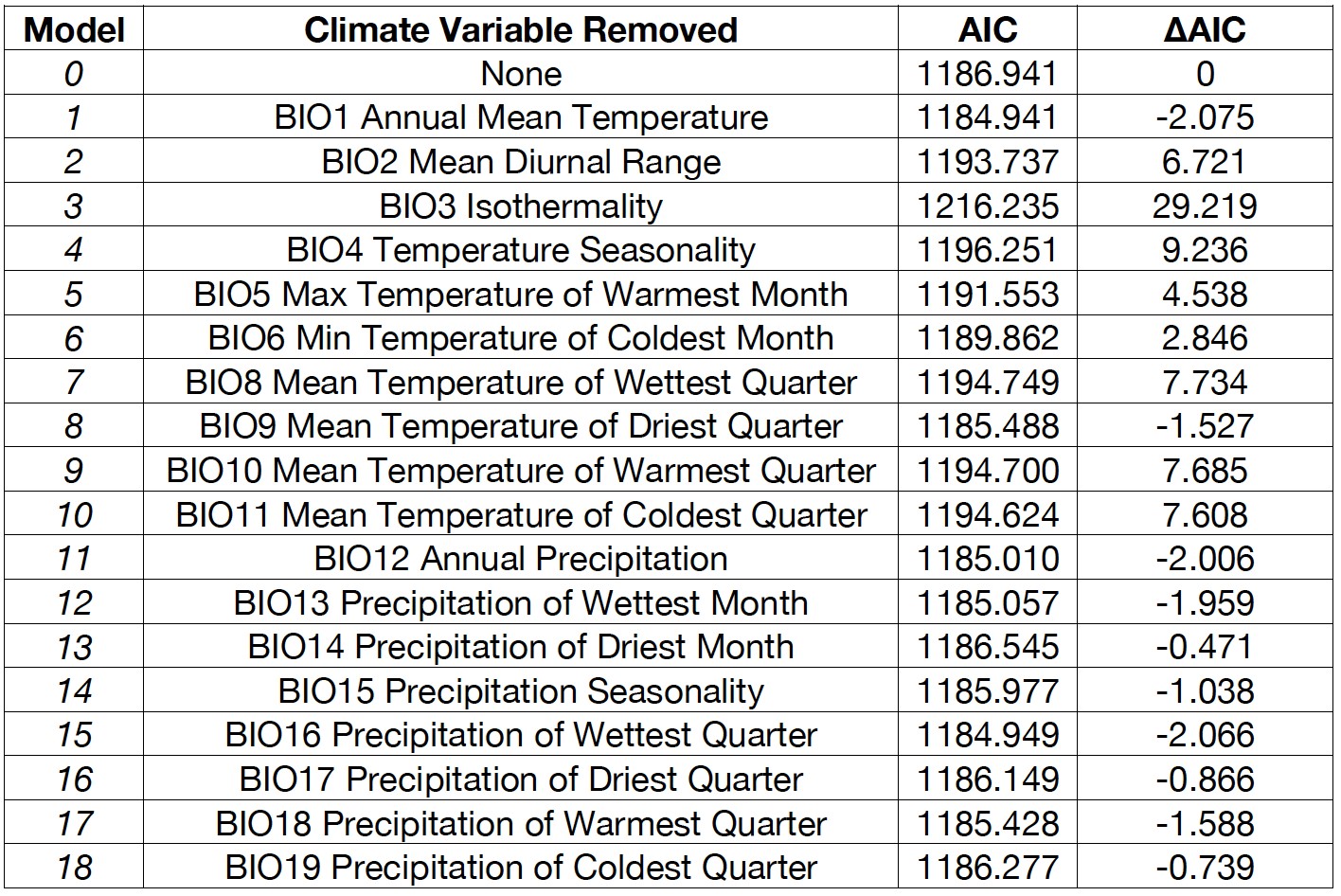

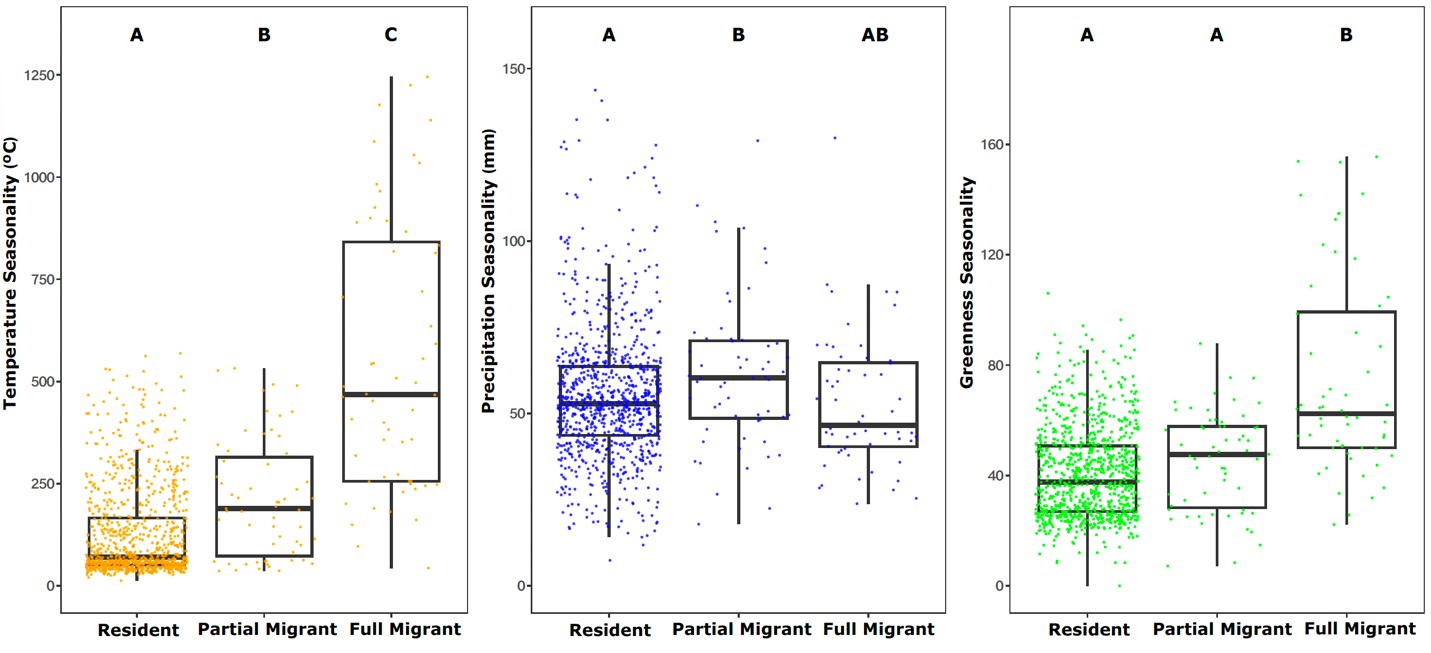


**Figure S1.** Temperature seasonality, precipitation seasonality, and greenness seasonality vary depending on migratory behavior using a distance-based coding. **LEFT:** Birds with different migratory strategies live in climates with different degrees of temperature seasonality. Temperature seasonality is significantly associated with migration, regardless of migratory behavior (resident p = 2.00E-16, partial migrant = 1.77E-06, full migrant p = 2.00E-16). Average temperature seasonality, in degrees Celsius, of breeding resident range is plotted on the vertical axis. **MIDDLE:** Resident and partially migratory birds live in climates with different degrees of precipitation seasonality. Fully migratory birds live in climates that are statistically similar to that of residents and partial migrants. Precipitation seasonality is significantly associated with migration in residents (p = 2.00E-16) and partial migrants (p = 0.0123). Precipitation seasonality is not significantly associated with fully migratory birds (p = 0.4198). Average precipitation seasonality, in millimeters, of each bird’s breeding-resident range is plotted on the vertical axis. **RIGHT:** Resident and partially migratory birds live in climates with statistically similar degrees of greenness seasonality. Fully migratory birds live in climates that are different from both residents and partial migrants. Greenness seasonality is significantly associated with migration, regardless of migratory behavior (resident p = 2.00E-16, partial migrant = 0.0435, full migrant p = 2.00E-16). Average greenness seasonality, unitless, of breeding-resident ranges is plotted on the vertical axis.


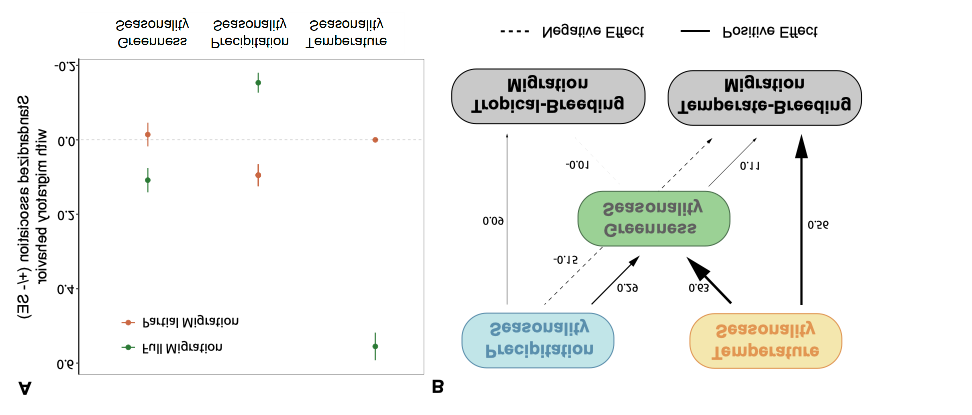


**Figure S2.** Phylogenetic path analysis with distance-based coding. **A.** Plot depicting the standardized associations (effect magnitude of greenness seasonality, precipitation seasonality, and temperature seasonality) with respect to partial and full migrants. Of the three climate variables, temperature seasonality has an overwhelmingly strong association (0.56) with migration in fully migratory birds. Precipitation seasonality and greenness seasonality have moderate negative (-0.15) and positive (0.11) relationships with migration in fully migratory birds, respectively. All three climate variables show weak relationships with migration in partially migratory birds (temperature seasonality: 0, precipitation seasonality: 0.09, greenness seasonality: -0.01). **B.** Phylogenetic path analysis illustrating the interactions between climate variables and different migratory behaviors. Thicker lines represent stronger associations. The direct association between temperature seasonality and migration in fully migratory birds is strong. The association between temperature seasonality and greenness seasonality is the strongest. The association between greenness seasonality and migration in partially migratory birds is the weakest.

**Table S2.** We examined the influence of climate change on suboscine species with different migratory strategies. The difference was calculated between historical and future seasonality values for two different Shared Socioeconomic Pathways, SSP 126 and SSP 370. This table summarizes the mean changes in temperature seasonality and precipitation seasonality by migratory strategy and Shared Socioeconomic Pathway.

|  | **Change in Temperature Seasonality (ºC)** | | **Change in Precipitation Seasonality (mm)** | |
| --- | --- | --- | --- | --- |
|  | *SSP 126* | *SSP 370* | *SSP 126* | *SSP 370* |
| Resident | -3.632 | -17.469 | 0.288 | -39.789 |
| Tropical-breeding Migrant | -4.345 | -16.948 | -0.250 | -8.496 |
| Temperate-breeding Migrant | -7.296 | -5.606 | -0.710 | -39.546 |


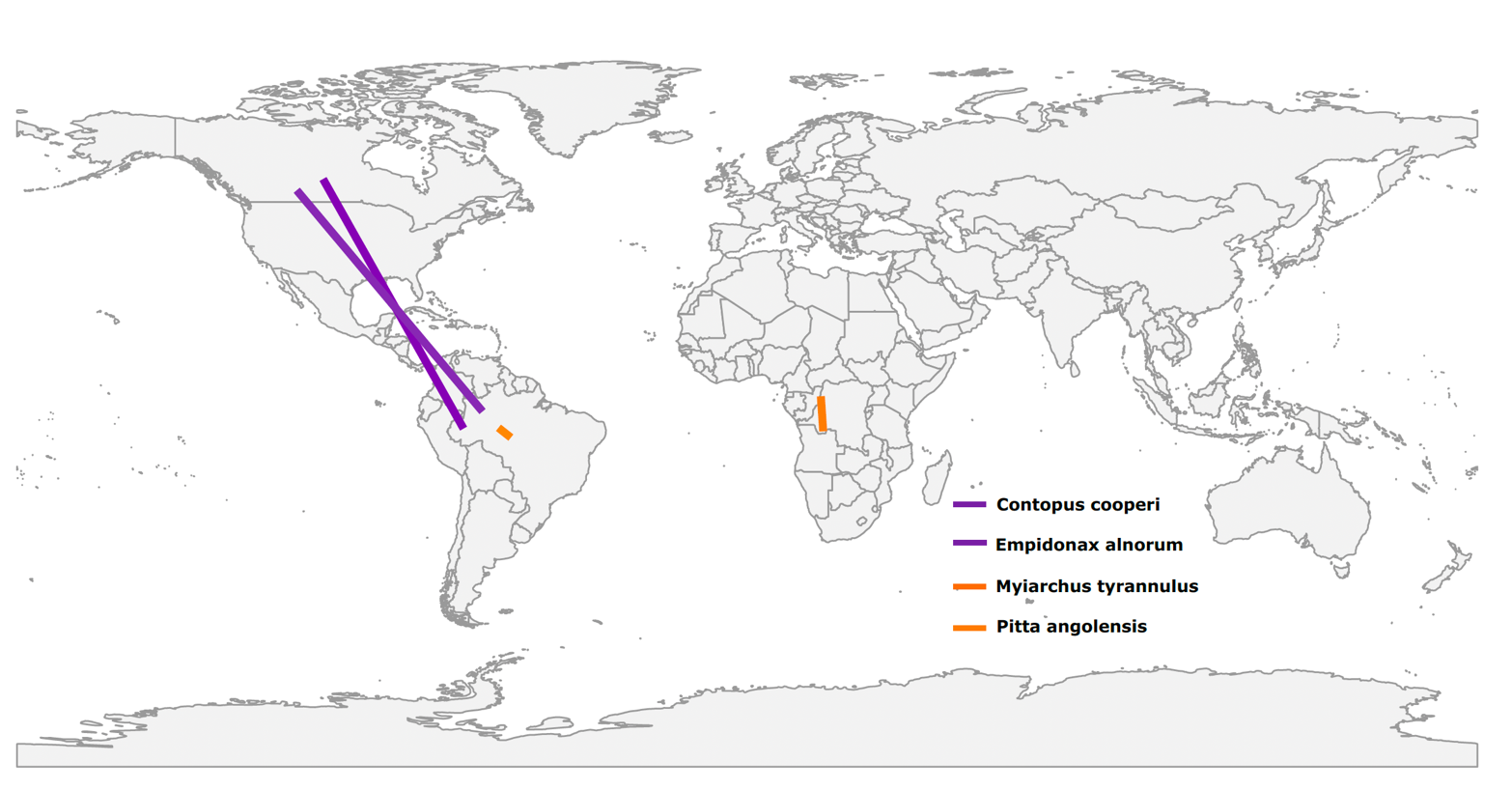


**Figure S3.** Migration routes of four focal species with different migratory strategies. Temperate-breeding migratory species, *Contopus cooperi* and *Empidonax alnorum*, are illustrated in purple. Tropical-breeding migratory species, *Myiarchus tyrannulus* and *Pitta angolensis*, are illustrated in orange. The Olive-sided Flycatcher (*C.cooperi*) and Alder Flycatcher (*E.alnorum*) breed as far north as western Canada then return to northern Brazil to winter each year, traveling approximately 7,500 kilometers one way. In contrast, the shorter-distance migrants undergo movement between their breeding and wintering grounds but stay within regions that experience more consistent, warmer climates, traveling less than 1,000 kilometers and often much less than this.
